# Exploring the interaction mechanisms of CD46/TREM1 and LC3B/ATG5 in the inflammation-cancer transformation of oral squamous cell carcinoma based on bioinformatics

**DOI:** 10.3389/fmolb.2025.1713632

**Published:** 2025-12-03

**Authors:** Huixian Xie, Yingjie Xu, Beibei Cong, Meihua Gao, Wanchun Wang

**Affiliations:** 1 School of Stomatology, Qingdao University, Qingdao, China; 2 Central Laboratory, Qingdao Stomatological Hospital Affiliated to Qingdao University, Qingdao, China; 3 Department of Oral Mucosa, Qingdao Stomatological Hospital Affiliated to Qingdao University, Qingdao, China

**Keywords:** CD46/TREM1, LC3B/ATG5, oral squamous cell carcinoma (OSCC), inflammation-cancer evolution, bioinformatics

## Abstract

**Objective:**

To investigate the molecular interaction patterns between CD46/TREM1 and LC3B/ATG5 in the development of oral squamous cell carcinoma (OSCC), providing novel targets for elucidating the mechanism of inflammatory-to-cancer progression and for the early diagnosis and treatment of OSCC.

**Methods:**

An oral inflammation-to-cancer progression animal model was established using 4-Nitroquinoline-N-oxide (4-NQO) drinking water and/or lipopolysaccharide (LPS). Clinical oral leukoplakia (OLK), OSCC, and adjacent non-cancerous tissues were collected. Immunohistochemistry assessed CD46, TREM1, LC3B, ATG5 protein expression and PI3K-AKT/TNF pathway alterations in animal and clinical tissues. Enzyme-Linked Immunosorbent Assay (ELISA) measured inflammatory cytokine levels in serum and saliva. High-throughput sequencing analyzed key pathways.

**Results:**

Immunohistochemistry revealed elevated CD46/TREM1 expression and reduced LC3B/ATG5 expression in OSCC tissues (*P* < 0.05). Serum levels of IL-6, IL-8, and GROα/CXCL1 progressively increased with advancing inflammation-to-cancer progression in rats, whereas salivary expression peaks occurred during the inflammatory phase. In human saliva and serum, TNF-α, IL-8, and IL-6 exhibited an increasing trend among healthy individuals, oral leukoplakia patients, and OSCC patients (*P* < 0.05). Transcriptome analysis revealed a significant increase in differentially expressed genes during the transformation from OLK to OSCC, predominantly downregulated genes. Among these, *Col4a6* and *Csf2* genes participated in inflammation-to-cancer progression by regulating the PI3K-Akt and TNF pathways.

**Conclusion:**

CD46 and TREM1 are highly expressed in OSCC and serve as key initiating factors in the progression from OLK to OSCC. Bioinformatics analysis identified critical candidate genes (*Col4a6*, *Csf2*) and pathways (PI3K-Akt, TNF) in inflammation-to-cancer conversion. Activation of the PI3K-AKT-mTOR pathway is associated with inhibited autophagy and malignant progression of OSCC. Additionally, inflammation-to-cancer transition is a core mechanism in the development of OSCC, with the tumor inflammatory microenvironment acting as a “promoter” in the progression from OLK to OSCC. This study provides novel insights into the molecular mechanisms and targeted therapies for OSCC, holding significant theoretical and clinical application value.

## 1 Introduction

Malignant tumors of the oral and maxillofacial region pose a serious challenge to global public health—these tumors not only cause functional impairment in patients but also maintain high mortality rates. Oral cancers are overwhelmingly dominated by oral squamous cell carcinoma (OSCC), which constitutes the vast majority (approximately 90%) of diagnosed cases (Sun et al., 2021). Driven by global population aging and lifestyle shifts, the incidence of OSCC shows a persistent upward trend, particularly pronounced in developing countries (Zhang et al., 2016). Well-established risk factors include tobacco use, alcohol consumption, chronic betel nut chewing, poor oral hygiene, genetic predisposition, and infections (Chaturvedi et al., 2022). In recent years, oral microbiota imbalance has gained significant attention as a novel risk factor (Yan et al., 2025). Research confirms that specific oral bacteria, such as *Fusobacterium* nucleatum, are closely associated with OSCC development (Yamamoto et al., 2023). Our research group previously conducted cellular-level studies revealing that CD46—referred to as a “Pathogen Magneto-Effect Molecule” (Liszewski and Atkinson, 2021)—and TREM1 (Li et al., 2024) (Triggering receptor expressed on myeloid cells-1), which amplifies microbe-mediated inflammation, are highly expressed in human OSCC cells and are associated with malignant proliferation and metastasis of OSCC(Liu et al., 2025). However, no reports on the molecular mechanisms of these two molecules have been published domestically or internationally to date. Notably, CD46 and TREM1 may collaboratively shape a tumor-promoting microenvironment: CD46 potentially mediates immunosuppression via T-cell regulation, while TREM1 drives sustained production of pro-inflammatory factors (e.g., TNF-α, IL-6) by amplifying NF-κB signaling. This chronic inflammatory state is closely linked to autophagy dysregulation, with the PI3K-AKT-mTOR pathway serving as a critical link (Rajendran et al., 2024; Kwack et al., 2025). We thus hypothesize that CD46/TREM1 may contribute to the inflammation-cancer transformation in OSCC by activating the PI3K-AKT-mTOR pathway and subsequently inhibiting autophagy, as indicated by downregulated LC3B/ATG5. Building on this foundation, we established a 4-Nitroquinoline-N-oxide (4-NQO)-induced rat model of inflammation-to-cancer progression in OSCC. Combined with clinical sample analysis, we comprehensively employed transcriptomic sequencing, immunohistochemistry (IHC), and Enzyme-Linked Immunosorbent Assay (ELISA). This study provides novel targets for elucidating the molecular mechanisms of OSCC and its early diagnosis and treatment, holding significant theoretical implications and clinical application value.

## 2 Materials and methods

### 2.1 Experimental animals and model establishment

Forty-two 6-week-old male Sprague-Dawley rats (200–220 g) were randomly allocated into seven groups (initial n = 6 per group) using a computer-generated random number sequence: normal control (NC), inflammation (INF), INF + lipopolysaccharide (LPS), oral leukoplakia (OLK), OLK + LPS, OSCC, and OSCC + LPS. The final group size of n = 4, which meets the minimum requirement for transcriptome sequencing, was achieved by including only the animals that survived to the scheduled experimental endpoint, following the exclusion of those that died unexpectedly during the 4-NQO modeling process. This study adhered to the ARRIVE 2.0 guidelines (see Supplementary Material). All animals were maintained under specific pathogen-free conditions with controlled temperature (20 °C–25 °C), humidity (40%–60%), and a 12-h light/dark cycle, and cage positions were routinely rotated. They were fed purified sterile drinking water and SPF-grade sterile solid animal feed. 4-NQO is a water-soluble quinoline carcinogen structurally similar to nitrosamines—carcinogenic derivatives of nicotine found in tobacco. Administered via drinking water, ensuring sustained contact with oral and upper gastrointestinal mucosa, effectively mimicking human long-term carcinogen exposure via smoking or tobacco chewing (Sur et al., 2018). LPS was used as a pro-inflammatory agent to accelerate and exacerbate the chronic inflammatory response induced by 4-NQO, modeling the contribution of persistent microbial infection or severe inflammation to oral carcinogenesis. A 0.004% 4-NQO drinking water regimen established the “oral inflammation-leukoplakia-squamous cell carcinoma” SD rat model. NC group: drank ultrapure water. 4-NQO-induced groups (INF, OLK, OSCC): drank 0.004% 4-NQO solution in a light-protected bottle. 4-NQO+LPS-induced groups (INF+LPS, OLK+LPS, OSCC+LPS): drank 0.004% 4-NQO solution in a light-protected bottle while receiving concurrent local LPS application. LPS (3 mg/mL in PBS)was applied topically to the dorsal tongue and entire oral mucosa using cotton swabs at regular intervals (3 times weekly) (Shiba et al., 2022). Weekly body weight measurements were taken; a weight loss exceeding 20% indicated deteriorating animal health (Steyn et al., 2023). Biweekly macroscopic oral examinations and photographic documentation were performed to observe mucosal changes, leukoplakia formation, and tumor development. Following deep anesthesia induced by an intraperitoneal injection of sodium pentobarbital (40 mg/kg), saliva was stimulated and collected via an injection of Pilocarpine Nitrate (5 mg/kg, MCE, HY-B1006) (Benarde et al., 1956). Subsequently, rats were euthanized by terminal cardiac puncture. Blood samples were collected and centrifuged to obtain serum. Saliva and serum samples were collected during normal, inflammatory, leukoplakia, and OSCC (56 samples in total) and stored at −20 °C. Tongue tissue samples were collected at different time points and grouped based on histopathological diagnosis. A total of 28 tissue samples were collected across the four stages. One portion was placed overnight in RNA preservation solution (AG, AG21015) at 4 °C before transfer to −80 °C storage. The other portion was fixed in 10% formalin and processed into paraffin sections.

### 2.2 Clinical sample collection

From July to December 2024, oral tissue samples were collected from 20 patients (12 males and 8 females, aged 31–67), including 7 with oral leukoplakia (OLK), 7 with oral squamous cell carcinoma (OSCC), and 6 with adjacent normal mucosa (serving as normal controls, NC). All diagnoses were clinically and histopathologically confirmed. A portion of the tissue samples was stored at −80 °C, while another portion was fixed and embedded in paraffin. Paired saliva and serum samples (40 samples in total) were obtained from the same 20 patients and stored at −20 °C.

### 2.3 Hematoxylin-eosin staining

The paraffin sections were sequentially subjected to deparaffinization to hydration, hematoxylin staining, eosin staining, dehydration, transparency, and mounting with neutral gum. After drying, the sections were observed under a microscope.

### 2.4 IHC analysis

Sections were baked at 60 °C for 1 h, dewaxed, and rehydrated before antigen retrieval (Tris-EDTA (×20)). After adding an appropriate amount of endogenous peroxidase blocker (PV-9001, Zhongshan Jinqiao, China), the corresponding primary antibody was added dropwise and incubated overnight at 4 °C. Primary antibodies used: CD46 (1:500, MCE, HY-P 80064), TREM1 (1:300, Bioss, bs-10306R), LC3B (1:100, MCE, HY-P 80742), ATG5 (1:600, Immunoway, YM8340). Subsequently, a rabbit two-step detection kit (PV-9001, Zhongshan Jinqiao, China) was used for reaction. DAB development was performed for 5 min, followed by hematoxylin counterstaining and neutral balsam mounting. A semi-quantitative H-Score (Wen et al., 2024) was applied to evaluate protein expression levels based on staining intensity, which was categorized and scored as 0 (negative), 1 (weak), 2 (moderate), or 3 (strong). The H-Score was derived from the formula: H-Score = (% weak × 1) + (% moderate × 2) + (% strong × 3), yielding a theoretical range of 0–300. H-Score assessment was performed in triplicate by two blinded independent observers with excellent inter-rater reliability (ICC = 0.89).

### 2.5 ELISA

Using ELISA kits (Elabscience, E-EL-R-0015, E-EL-R-0003, E-EL-R-2856, E-EL-H6156, E-EL-H6008) to measure serum and saliva IL-6, TNF-α, and IL-8/CXCL1 levels in rat and clinical samples. Upon addition of the chromogenic substrate (TMB), the reaction solution initially turns blue due to horseradish peroxidase catalysis. After adding the stop solution, the color changes from blue to yellow. Following this, the absorbance (optical density, OD) at 450 nm was determined with an ELISA microplate reader. Specific concentrations of inflammatory cytokines in the samples are calculated by constructing a standard curve. All samples were analyzed in triplicate with three independent experimental repeats.

### 2.6 Transcriptome sequencing and bioinformatics analysis

#### 2.6.1 RNA extraction and quality control

Total RNA extracted from tissues underwent quality control using a Nanodrop2000 (concentration/purity), agarose gel electrophoresis (integrity), and an Agilent 2100 system (RIN score). RNA for library construction must meet the following criteria: total content ≥1 μg, concentration ≥35 ng/μL, OD260/280 ratio ≥1.8, and OD260/230 ratio ≥1.0.

#### 2.6.2 mRNA capture via oligo dT

mRNA was isolated from total RNA employing Oligo (dT) magnetic beads, which selectively bind to the polyA tails of eukaryotic mRNAs via complementary base pairing, to facilitate transcriptome sequencing.

#### 2.6.3 mRNA fragmentation

Since the Illumina NovaSeq 6000 sequencing platform is suitable for short-read sequencing, while enriched mRNA typically consists of intact long chains averaging several thousand bases in length, random fragmentation is required. mRNA was randomly sheared using a fragmentation buffer. Subsequently, fragments of the desired size (∼300 bp) were isolated by magnetic bead purification for downstream library construction.

#### 2.6.4 cDNA synthesis

The first strand of cDNA was synthesized using purified mRNA as a template, random hexamers as primers, and reverse transcriptase for catalysis. The second strand is subsequently synthesized, yielding double-stranded cDNA products.

#### 2.6.5 Adapter ligation

To allow adapter ligation, the sticky ends of ds cDNA were blunted with a repair enzyme mix and then tailed with a single adenine (A) nucleotide at the 3′ ends.

#### 2.6.6 Illumina sequencing

Perform the following steps on the constructed library: First, conduct PCR amplification (15 cycles), then recover the target fragment via 2% agarose gel electrophoresis; Quantify the library using the TBS380 (Picogreen) fluorescent quantification method and mix samples at corresponding ratios; Cluster generation was performed on the cBot system via bridge PCR, followed by paired-end sequencing (2 × 150 bp) on the Illumina platform.

### 2.7 Differentially expression genes (DEGs) screening

Raw sequencing data underwent quality control using FastQC software and were aligned to the reference genome with HISAT2 software (Wang et al., 2024). Gene expression levels were estimated using StringTie. Using raw data as input, DESeq2 is employed for pre-screening in differential expression analysis to remove genes with insignificant expression, followed by formal screening of DEGs in each group. Comparison groups included: INF vs. NC, OLK vs. INF, OSCC vs. OLK (animal experiments); OLK vs. NC, OSCC vs. OLK (clinical samples). P-values were corrected for multiple testing with the Benjamini–Hochberg method, resulting in adjusted *P*-values (*P* adj).

Pre-screening criteria were as follows: (1) Expression level was zero in all samples; (2) Expression level was below 10 in at least three samples.

The screening threshold for DEGs was set as: |log_2_(FoldChange)| > 2 and *P* adj <0.05.

### 2.8 Validation of PI3K-Akt and TNF signaling pathway-related proteins via immunohistochemistry

Methods were identical to those described in Section 2.4. Primary antibodies used included: TNF-α (1:100, MCE, HY-P 80914), P-PI3K (1:100, MCE, HY-P 80846), P-AKT (1:600, MCE, HY-P 80276), and P-MTOR (1:100, BOSTER, BM4840). P-AKT and P-MTOR serve as marker molecules for PI3K-Akt pathway activation and its downstream effects, respectively, while TNF-α is a core factor in the inflammatory pathway.

### 2.9 Statistical analysis

The threshold for statistical significance was set at *P* < 0.05. All analyses were carried out in GraphPad Prism 8.0, with results reported as mean ± SD. Data normality and variance homogeneity were verified prior to analysis. Effect sizes (Cohen’s d) with 95% confidence intervals (95% CI) are reported for key comparisons (Supplementary Table S1). Specifically, we employed the Student’s t-test to compare two groups and one-way ANOVA with Tukey’s *post hoc* test for comparisons across multiple groups. Pearson correlation analysis was performed on individual animal (n = 16) and human (n = 20) H-Scores.

## 3 Results

### 3.1 Animal experiment results

#### 3.1.1 Successful establishment of a rat model of inflammation-to-cancer progression in OSCC induced by 4-NQO

**FIGURE 1:**
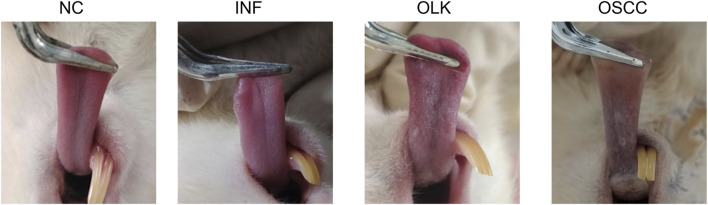
Symptomatic manifestations of the tongue in SD rats at four stages: normal (NC), inflammatory (INF), leukoplakia (OLK), and squamous cell carcinoma (OSCC).

Normal SD rats exhibit a soft tongue with a pink, elastic dorsal mucosa. In the 4-NQO group, mild inflammation, erosion, or ulceration appeared on the dorsal tongue mucosa between weeks 6 and 8. White patches were visible on the dorsal tongue mucosa between weeks 8 and 16. Between weeks 16 and 22, verrucous or ulcerative carcinomas were observed on the dorsal tongue mucosa, predominantly near the dorsal tongue sulcus. In the 4-NQO+LPS-induced group, inflammation, leukoplakia, and tumor formation occurred earlier than in the 4-NQO group (5–7 days). This indicates that LPS, as a pro-inflammatory agent, accelerates the oral carcinogenesis process induced by 4-NQO (Figure 1).

#### 3.1.2 Histopathological observations of oral tissue in rats

**FIGURE 2:**
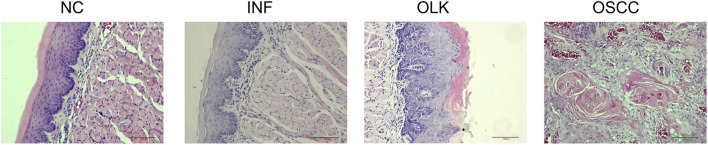
Hematoxylin and eosin staining (×20) of SD rats at four stages: normal (NC), inflammation (INF), leukoplakia (OLK), and squamous cell carcinoma (OSCC).

The NC group exhibited intact tissue structure without abnormal hyperplasia or malignant features. The most characteristic change in the INF group was dense chronic inflammatory cell infiltration in the lamina propria, predominantly lymphocytes, exhibiting band-like infiltration closely adhering to the epithelial-connective tissue junction. The OLK group showed hyperkeratinization of the epithelial layer with abnormal epithelial proliferation, including architectural disorganization, cellular atypia, increased mitotic figures, disrupted cellular maturation, and layering disorder. Chronic inflammatory cell infiltration (lymphocytes, plasma cells) of varying degrees was observed in the superficial lamina propria. Tumor cell nests were present in the OSCC group: cells exhibited marked atypia with variable size and morphology, deeply stained nuclei with prominent nucleoli, and numerous mitotic figures. Intercellular bridges were absent. Keratin pearls were visible in the center of the cancer nests (Figure 2).

#### 3.1.3 Expression of CD46, TREM1, LC3B, and ATG5 proteins in rat oral tissue

**FIGURE 3:**
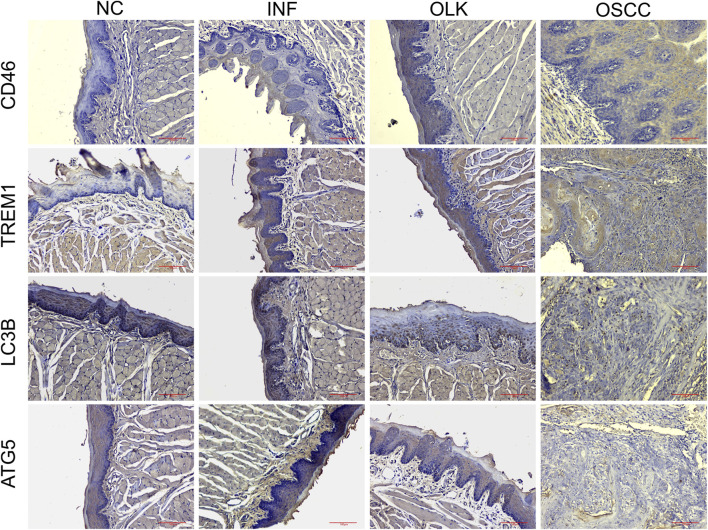
Expression of CD46/TREM1 and LC3B/ATG5 in four stages of SD rats: normal (NC), inflammatory (INF), leukoplakia (OLK), and squamous cell carcinoma (OSCC) (×20).

In normal oral mucosa, CD46 is expressed at low to moderate levels, while TREM1 is scarcely expressed. In inflammatory tissue, CD46 expression increases with enhanced membrane staining, and TREM1 is significantly upregulated. A large number of inflammatory cells with mononuclear/polymorphonuclear morphology, along with some epithelial cells, exhibit high expression of TREM1. In both leukoplakia and OSCC tissues, tumor cells exhibited diffuse, strong cytoplasmic/membrane staining. CD46 and TREM1 were highly expressed, while LC3B and ATG5 were expressed at low levels, with these differences being more pronounced in the OSCC group (Figure 3).

H-Score semi-quantitative results (Table 1) were consistent with the aforementioned morphological observations. Compared to normal tissue, CD46 and TREM1 scores were significantly elevated in inflammatory, leukoplakia, and OSCC tissues (*P* < 0.05). Conversely, LC3B and ATG5 scores were significantly reduced (*P* < 0.05).

**TABLE 1:** Immunohistochemical scoring (H-Score) of CD46, TREM1, LC3B, and ATG5 in rat oral tissue.

Group	CD46 (Mean ± SD)	TREM1 (Mean ± SD)	LC3B (Mean ± SD)	ATG5 (Mean ± SD)
NC	80.5 ± 15.2	75.3 ± 14.8	280.5 ± 25.4	265.8 ± 24.1
INF	175.6 ± 28.4	220.8 ± 35.1	160.2 ± 22.3	150.5 ± 20.7
OLK	210.3 ± 32.7	235.6 ± 30.9	120.4 ± 18.9	110.8 ± 17.5
OSCC	295.8 ± 30.5	285.4 ± 45.2	85.3 ± 16.2	70.1 ± 15.8

#### 3.1.4 Elevated levels of inflammatory cytokines in rat serum and saliva

**FIGURE 4:**
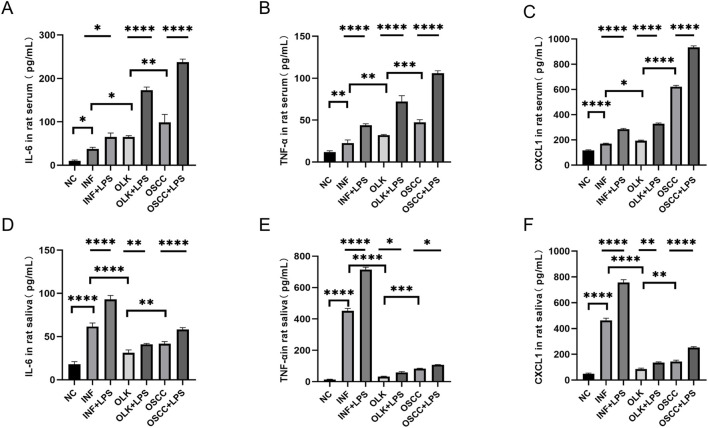
Levels of IL-6, IL-8, and GROα/CXCL1 in saliva and serum of SD rats ( * indicates *P* < 0.05, ** indicates *P* < 0.01, *** indicates *P* < 0.001, **** indicates *P* < 0.0001).

The 4-nitroquinoline-N-oxide (4-NQO) drinking water method established an SD rat model progressing through four stages: normal, inflammatory, leukoplakia, and squamous cell carcinoma. In serum, IL-6, IL-8, and GROα/CXCL1 levels increased sequentially (*P* < 0.05). In saliva, IL-6, IL-8, and GROα/CXCL1 reached peak concentrations during the inflammatory stage (*P* < 0.05); the 4-NQO+LPS group exhibited significantly higher inflammatory factors than the 4-NQO group (*P* < 0.05). These findings indicate that inflammation-to-cancer transition is a critical process in oral carcinogenesis, with chronic inflammation acting as a “promoter” in this pathway (Figures 4A–F).

#### 3.1.5 Transcriptome sequencing revealed dynamic changes in gene expression profiles during inflammation-to-cancer progression

Transcriptome analysis indicated progressively worsening gene dysregulation during oral cancer development. To delineate the molecular events at inflammation onset, we first compared the INF and NC groups. Volcano plot analysis (set with screening criteria *P*-value <0.05 and |log_2_(FoldChange)| > 2) identified DEGs between groups: 620 upregulated and 189 downregulated in INF versus NC; 740 upregulated and 533 downregulated in OLK versus INF; and 1,597 upregulated and 2,365 downregulated in OSCC versus OLK. During progression from precancerous lesions (OLK) to malignant tumors (OSCC), the total number of DEGs increased by 3.1-fold compared to the early stage, with downregulated genes outnumbering upregulated ones. Clustering analysis revealed strong homogeneity within each group and distinct differences between groups (Supplementary Figure S1).

**FIGURE 5:**
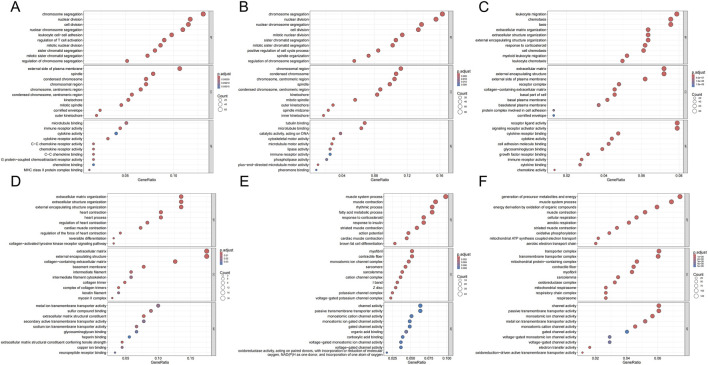
GO enrichment bubble plots for DEGs across groups. **(A,D)** GO terms enriched for genes up- **(A)** and downregulated **(D)** in INF compared to NC. **(B,E)** GO terms enriched for genes up- **(B)** and downregulated **(E)** in OLK compared to INF. **(C,F)** GO terms enriched for genes up- **(C)** and downregulated **(F)** in OSCC compared to OLK. For all subfigures, the bubble size indicates the number of genes associated with the GO term, and the color gradient (blue to red) represents the statistical significance of the enrichment. GO categories are labeled as Biological Process (BP), Cellular Component (CC), and Molecular Function (MF).

From the normal to inflammatory stages, upregulated genes were significantly enriched in cell proliferation-related functions (e.g., chromosome segregation, mitosis) and immune response-related functions (e.g., immune receptor activity, cytokine receptor activity) (Figure 5A). This indicated compensatory proliferation and intense immune cell activation in response to tissue injury. Downregulation primarily manifested as suppression of extracellular matrix (ECM) organization and structural functions (Figure 5D), suggesting that normal tissue architecture was remodeled or disrupted during the inflammatory response. From the inflammatory to the leukoplakia stage, upregulated genes were significantly enriched in cell division and chromosome-related functions (e.g., nuclear chromosome segregation, chromosome centromeres) (Figure 5B), indicating intensified abnormal cell proliferation accompanied by significant genomic instability. Significant downregulation of muscle contraction and ion channel-related functions reflected tissue-specific functional loss during the precancerous stage (Figure 5E). From the leukoplakia to OSCC stage, upregulated genes were significantly enriched in leukocyte migration, chemotaxis, and cytokine signaling (Figure 5C), indicating that malignant tumor cells actively recruit immune cells and construct an immunosuppressive tumor microenvironment to support their growth. Energy metabolism and ion transmembrane transport functions were broadly suppressed (Figure 5F), revealing that tumor cells underwent typical metabolic reprogramming (Warburg effect) to adapt to rapid proliferation, abandoning efficient oxidative phosphorylation.

**FIGURE 6:**
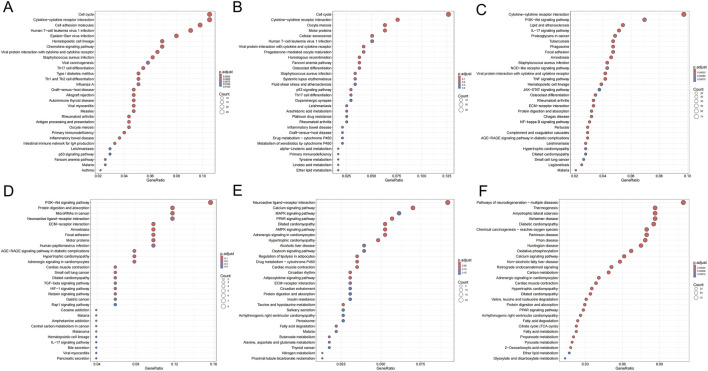
KEGG enrichment bubble plots for DEGs across groups. **(A,D)** Pathways enriched for genes up- **(A)** and downregulated **(D)** in INF compared to NC. **(B,E)** Pathways enriched for genes up- **(B)** and downregulated **(E)** in OLK compared to INF. **(C,F)** Pathways enriched for genes up- **(C)** and downregulated **(F)** in OSCC compared to OLK. For all subfigures, the bubble size represents the number of genes mapped to the KEGG pathway, and the color intensity (blue to red) corresponds to the statistical significance of the enrichment.

As shown in Figures 6A–F, KEGG pathway analysis dynamically revealed the core driving force behind oral cancer progression: chronic inflammation persistently activates proliferation signals and disrupts homeostasis pathways, ultimately leading to a malignant phenotype characterized by immune-metabolic dysregulation. First, the PI3K-Akt pathway reversed from early downregulation to late upregulation, representing the “awakening” of a classic oncogenic signal. Second, pathways maintaining cellular homeostasis—such as calcium signaling and MAPK—remained persistently inactivated during precancerous lesions. Third, late-stage-specific metabolic dysregulation markers (thermogenesis suppression) emerged. Abnormalities in the “cytokine-cytokine receptor interaction” pathway were observed across all three stages. The consistent presence of this pathway throughout suggested chronic inflammation plays a pivotal role as a driving factor.

From oral leukoplakia to squamous cell carcinoma, TNF signaling was upregulated, consistent with ELISA results showing progressive increases in IL-6, IL-8, and GROα/CXCL1 in rat serum, indicating the critical role of the inflammatory microenvironment in carcinogenesis. Concurrently, the PI3K-AKT signaling pathway was activated, consistent with immunohistochemical findings. When the PI3K-AKT-mTOR pathway was active, autophagy was suppressed, as evidenced by reduced LC3B lipidation and decreased formation of the ATG5-ATG12 complex.

#### 3.1.6 Activation of TNF and PI3K-AKT signaling pathways in rat oral carcinogenesis

**FIGURE 7:**
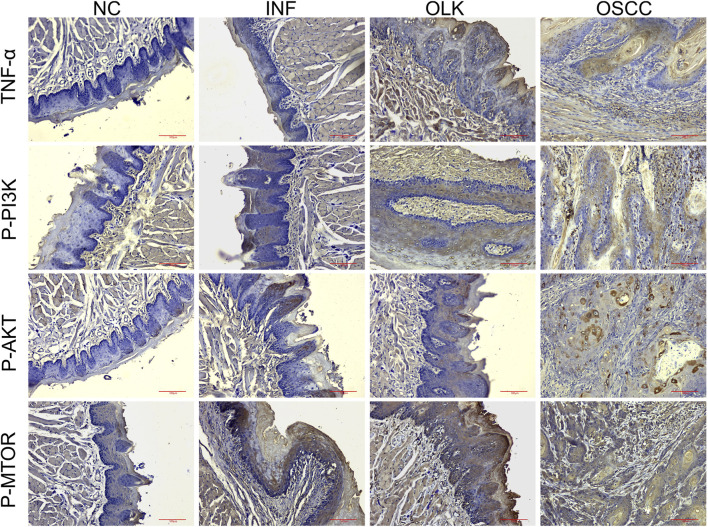
Expression of TNF-α, P-PI3K, P-AKT, and P-MTOR proteins during the carcinogenesis process in SD rats (×20).

P-PI3K, P-AKT, and P-MTOR proteins were primarily localized in the cytoplasm/membrane, while TNF-α was expressed in keratinocytes, infiltrating inflammatory cells (e.g., macrophages, lymphocytes), and some cancer cells. Figure 7 immunohistochemical result clearly demonstrated that the expression intensity and activation levels of proteins associated with the TNF and PI3K-AKT signaling pathways progressively increased with disease progression. The critical activation observed during the leukoplakia stage suggested their pivotal role in mediating malignant transformation. The quantitative H-Score data for P-PI3K, P-AKT, P-MTOR, and TNF-α proteins in rat tissues are provided in Supplementary Table S3.

#### 3.1.7 Correlation of CD46/TREM1 with PI3K-AKT-mTOR and autophagy markers

Correlation analysis of individual rat H-Scores (n = 16) showed CD46/TREM1 expression was positively correlated with PI3K-AKT-mTOR pathway activation (P-PI3K, P-AKT, P-MTOR), and the pathway components were negatively correlated with LC3B/ATG5 levels (all |*r*| > 0.83, *P* < 0.001; Supplementary Table S3).

#### 3.1.8 Screening of key DEGs in inflammation-cancer progression

Based on the set threshold (|log_2_(FoldChange)| > 2 and *P* adj <0.05), we identified multiple significantly DEGs in the PI3K-Akt and TNF signaling pathways. Among extracellular matrix (ECM)-related genes, *Lama1* showed an upregulation trend, while *Col4a6* was markedly downregulated. Concurrently, two genes encoding neurotrophic factor receptors with tumor-suppressing effects—*Ntrk1* and *Erbb4*—also exhibited markedly reduced expression levels. Notably, multiple genes associated with pro-inflammatory promotion showed significantly elevated expression, such as granulocyte-macrophage colony-stimulating factor (*Csf2*) and chemokines *Ccl12* and *Ccl20*. Furthermore, the stress-activated protein kinase gene *Mapk12* exhibited downregulation. Collectively, these genes are involved in functions including extracellular matrix remodeling, inflammatory response regulation, and cellular stress responses.

### 3.2 Clinical trial results

#### 3.2.1 Expression of CD46, TREM1, LC3B, and ATG5 proteins in human oral tissue

**FIGURE 8:**
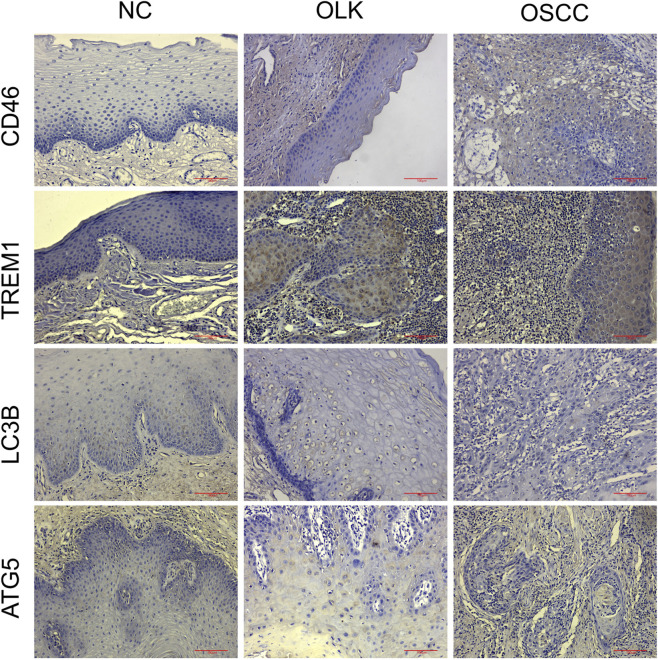
Expression of CD46/TREM1 and LC3B/ATG5 in human normal (NC), leukoplakia (OLK), and squamous cell carcinoma (OSCC) tissues (×20).

In normal oral mucosa, CD46 is expressed at low to moderate levels on epithelial cell membranes, while TREM1 is scarcely expressed on epithelial cells. In oral leukoplakia, CD46 expression is upregulated, membrane expression is enhanced in areas of abnormal epithelial hyperplasia, TREM1 expression is elevated, inflammatory cell infiltration is present, and LC3B/ATG5 expression is low. In oral squamous cell carcinoma (OSCC), CD46 shows markedly high expression with diffuse strong positivity on tumor cell membranes/cytoplasm, TREM1 is overexpressed, and LC3B/ATG5 expression is low (Figure 8).

H-Score semi-quantitative analysis (Table 2) revealed that compared to normal tissue, both CD46 and TREM1 expression scores were significantly elevated in leukoplakia and OSCC tissues (*P* < 0.05), peaking in OSCC. Conversely, LC3B and ATG5 scores decreased significantly with increasing malignancy (*P* < 0.05).

**TABLE 2:** Immunohistochemical scoring (H-Score) of CD46, TREM1, LC3B, and ATG5 in human oral tissue.

Group	CD46 (Mean ± SD)	TREM1 (Mean ± SD)	LC3B (Mean ± SD)	ATG5 (Mean ± SD)
NC	85.0 ± 12.5	78.0 ± 15.5	290.0 ± 22.0	275.0 ± 23.0
OLK	190.0 ± 26.0	210.0 ± 32.0	150.0 ± 20.0	140.0 ± 19.0
OSCC	290.0 ± 35.0	295.0 ± 42.0	90.0 ± 15.0	75.0 ± 14.0

#### 3.2.2 Elevated inflammatory cytokine levels in saliva and serum of oral leukoplakia and OSCC patients

**FIGURE 9:**
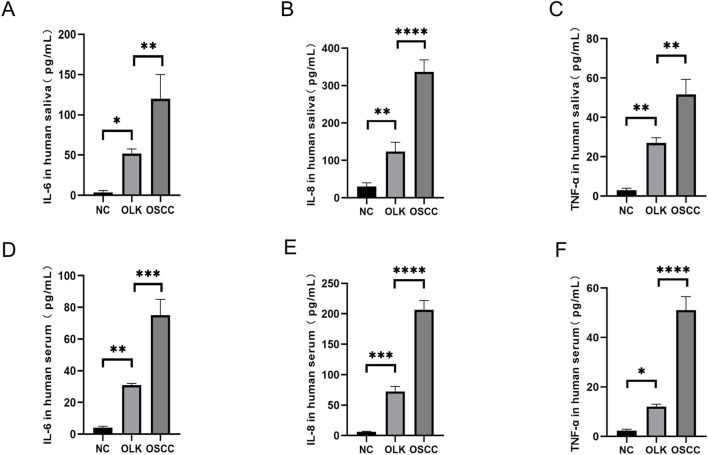
Levels of TNF-α, IL-8, and IL-6 in human saliva and serum ( * indicates *P* < 0.05, ** indicates *P* < 0.01, *** indicates *P* < 0.001, **** indicates *P* < 0.0001).

The concentrations of TNF-α, IL-8, and IL-6 in human saliva and serum progressively increased from healthy individuals to oral leukoplakia patients and finally to oral squamous cell carcinoma (OSCC) patients (*P* < 0.05). This clearly demonstrates that chronic inflammation plays a central role in promoting the carcinogenic process of oral mucosa. These cytokines constitute a critical “inflammatory microenvironment” that accelerates the progression from precancerous lesions (leukoplakia) to malignant tumors (OSCC), delineating a distinct “inflammation-to-cancer” pathway: various stimuli induce chronic inflammation in the oral mucosa, leading to elevated levels of TNF-α, IL-6, IL-8, and other factors that disrupt immune homeostasis. Epithelial cells undergo abnormal proliferation, while IL-6 and TNF-α activate the inflammation-to-cancer pathway, ultimately driving cellular carcinogenesis (Figures 9A–F).

#### 3.2.3 Transcriptomic analysis of clinical samples confirms malignant transformation characteristics from leukoplakia to OSCC

Based on (*P* adj <0.05, |log_2_(FoldChange)| > 1), results showed that 721 upregulated and 446 downregulated in OLK versus NC, and that 827 upregulated and1,538 downregulated in OSCC versus OLK. Thus, the total number of differentially expressed genes increased twofold during the malignant transformation from leukoplakia, with most genes showing significant downregulation. As shown in the figure, all sample groups demonstrated intra-group consistency and inter-group differences (Supplementary Figure S2).

**FIGURE 10:**
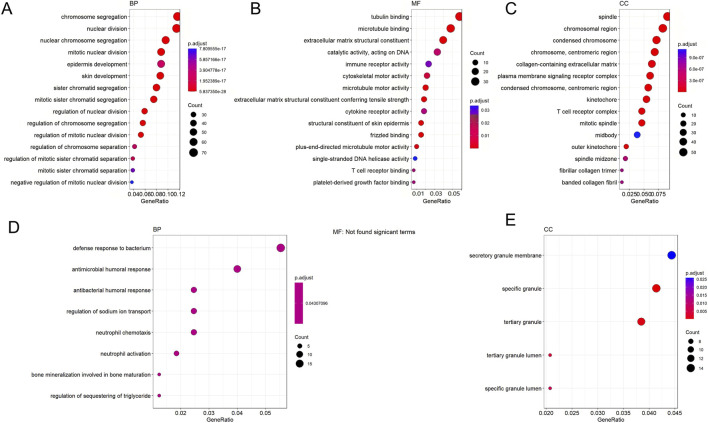
GO enrichment bubble plot of DEGs between NC and OLK (the latter as control). Figure **(A–C)** shows the enrichment plot for downregulated genes, and Figure **(D,E)** shows the enrichment plot for upregulated genes. The size of the bubble corresponds to the number of genes associated with the GO term, and the color intensity (blue to red) represents the level of enrichment significance.

**FIGURE 11:**
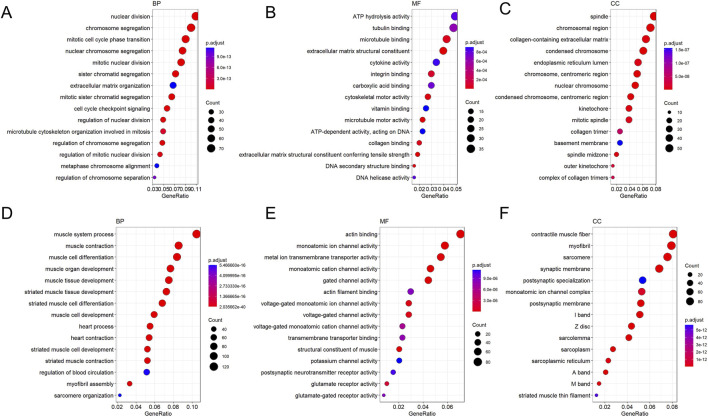
GO enrichment bubble plot of DEGs between OLK and OSCC (the latter as control). Figure **(A–C)** shows the enrichment plot for downregulated genes, and Figure **(D–F)** shows the enrichment plot for upregulated genes. The size of the bubble corresponds to the number of genes associated with the GO term, and the color intensity (blue to red) represents the level of enrichment significance.

GO functional enrichment analysis revealed that the most prominent feature from normal to leukoplakia stages was the comprehensive activation of cell division-related pathways (chromosome segregation, mitosis, etc.) accompanied by suppression of immune defense functions (downregulation of antimicrobial responses) (Figures 10A–E). From the leukoplakia to oral cancer stage, cell division-related pathways (mitosis, chromosome segregation) continued to intensify. This was accompanied by activation of signaling pathways associated with ATP hydrolysis activity (energy provision) and collagen-containing extracellular matrix (ECM). Malignant tumor cells maintained rapid proliferation, while tumor matrix remodeling led to OSCC invasion and metastasis (Figures 11A–F).

**FIGURE 12:**
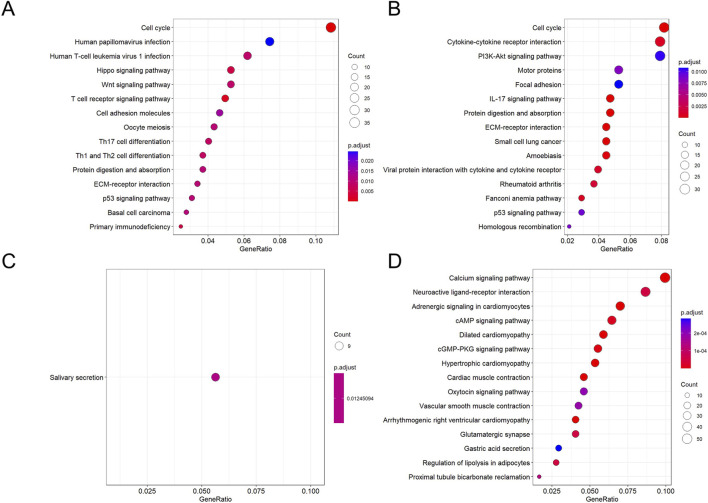
KEGG enrichment bubble plots for DEGs across groups. **(A,C)** Pathways enriched for genes down- **(A)** and upregulated **(C)** in NC compared to OLK. **(B,D)** Pathways enriched for genes down- **(B)** and upregulated **(D)** in OLK compared to OSCC. For all subfigures, the bubble size represents the number of genes mapped to the KEGG pathway, and the color intensity (blue to red) corresponds to the statistical significance of the enrichment.

During the oral leukoplakia stage, key developmental signaling pathways such as Hippo and Wnt are activated (Figure 12A), indicating disrupted regulatory mechanisms governing cell growth and differentiation. Concurrently, normal tissue functions like salivary secretion exhibit progressive decline (Figure 12C). As the lesion progresses toward squamous cell carcinoma, the upregulation of the classic pro-survival PI3K-Akt pathway provides cancer cells with sustained proliferative drive, anti-apoptotic capacity, and metabolic reprogramming support. Furthermore, the activation of cytokine-receptor interactions, actin cytoskeleton regulation, and focal adhesion signaling pathways indicates a restructuring of the tumor immune microenvironment (Figure 12B), while cancer cells acquire enhanced migratory and invasive properties. Conversely, key physiological signaling pathways such as calcium signaling, cAMP signaling, and neurotransmitter-receptor interactions are generally suppressed (Figure 12D). This indicates that in highly malignant tumors, the signaling capacity required for cells to maintain basic functions and internal homeostasis has largely collapsed.

#### 3.2.4 The TNF and PI3K-AKT signaling pathway are closely associated with the progression of oral cancer

**FIGURE 13:**
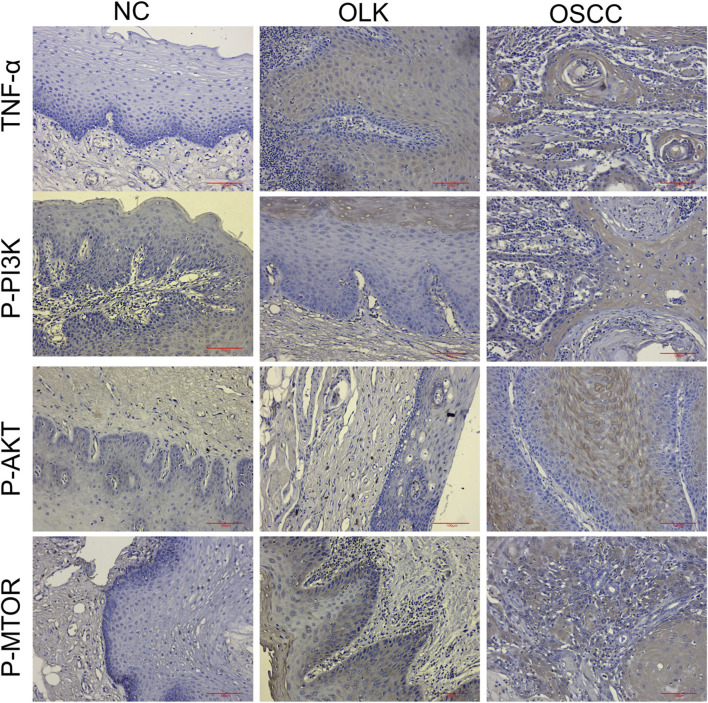
Expression of TNF-α, P-PI3K, P-AKT, and P-MTOR proteins during human oral carcinogenesis (×20).

As shown in Figure 13, within the PI3K-AKT signaling pathway, the evolution of phosphoprotein expression from absent/weak (normal) to focal enhancement (leukoplakia, especially in areas of dysplasia) to diffuse strong positivity (cancer) indicates progressively increasing pathway activity. In the TNF signaling pathway, TNF-α expression transitions from minimal expression (normal) to co-expression by epithelial and inflammatory cells (leukoplakia) to strong expression by cancer cells themselves with dense inflammatory infiltration (cancer). This reveals the evolutionary process of the inflammatory microenvironment shifting from a “participatory” to a “dominant” role. The corresponding H-Score data for human tissues are available in Supplementary Table S3.

#### 3.2.5 Correlation of CD46/TREM1 with PI3K-AKT-mTOR and autophagy in human tissues

Analysis of patient samples (n = 20) confirmed the same correlation pattern: positive between CD46/TREM1 and the PI3K-AKT-mTOR pathway, and negative between the pathway and LC3B/ATG5 (all |*r*| > 0.83, *P* < 0.001; Supplementary Table S3).

#### 3.2.6 Screening of key DEGs in clinical samples

Notably, *COL4A6* exhibited an upward trend in expression within human oral cancer tissues, starkly contrasting with the downregulation observed in rat models. Simultaneously, multiple genes encoding distinct functions also exhibited enhanced expression, such as those encoding growth factors (e.g., *OSM*, *ARTN*, and *FGF5*), G protein subunits (*GNGT1*), and laminin (e.g., *LAMA3*, *LAMB3*, and *LAMC2*). Regarding the inflammatory microenvironment, extensive activation of multiple related genes was observed, including cytokines (*CSF2*, *CSF3*, and *IL1B*), their corresponding receptors (*CSF3R*), and chemokines (e.g., *CXCL3*, *CXCL5*, and *CXCL6*). Additionally, elevated expression of matrix metalloproteinase genes *MMP 3* and *MMP 9* was observed, while *CXCL1* expression was downregulated. Collectively, these alterations shape a microenvironment conducive to tumor proliferation, invasion, and immunomodulation.

## 4 Discussion

In recent years, the standard treatment for oral cancer has been a comprehensive approach combining surgical resection with radiotherapy and chemotherapy (Polverini and Lingen, 2019). However, the therapeutic efficacy of this regimen remains inadequate: patients exhibit a high probability of postoperative recurrence, and when the disease progresses to the stage of distant metastasis, no effective control methods currently exist. Although new treatment approaches such as targeted therapy and immunotherapy (Dutta et al., 2022) have emerged in recent years, most remain in the clinical exploration phase, with efficacy yet to reach a stable state. OSCC severely impacts patient survival and quality of life. Due to its location, oral function is highly susceptible to significant impairment, manifesting as difficulties in chewing, swallowing, and speaking, alongside potential facial disfigurement (Wu et al., 2025). These issues further lead to communication barriers, inadequate nutritional intake, and social-psychological adaptation challenges. Collectively, these factors make OSCC a multidimensional public health challenge requiring comprehensive measures spanning prevention, early diagnosis, and innovative treatment approaches.

This study demonstrates that CD46/TREM1 is highly expressed in OSCC, while LC3B/ATG5 exhibits low expression in OSCC. As a complement regulatory protein, CD46 prevents complement membrane attack complex (MAC) formation by degrading C3b/C4b, thereby protecting tumor cells from complement-mediated lysis. It may also indirectly suppress antitumor immunity by regulating T cell function (Liszewski and Atkinson, 2021). TREM1 highly expressed in myeloid cells, maintains the tumor inflammatory microenvironment via the NF-κB/IL-6/STAT3 pathway; it may also directly activate pro-survival signals within tumor cells (Ren et al., 2024) (e.g., PI3K-AKT). Meanwhile, low expression of LC3B/ATG5 (autophagy defect): The LC3B and ATG5-ATG12 complex are key markers for autophagosome formation (Romanov et al., 2012), and their low expression indicates suppression of autophagy-mediated apoptosis. RNA sequencing results indicate that pathways closely associated with the progression of oral leukoplakia to carcinoma are “PI3K-Akt” and “TNF.” The PI3K-AKT-mTOR pathway is a negative regulatory pathway of autophagy. LC3B (microtubule-associated protein 1 light chain 3 beta) serves as a marker for autophagosome membrane formation, with its lipidation (conversion from LC3B-I to LC3B-II) dependent on the ATG5-ATG12 complex (involved in autophagosome elongation). When the PI3K-AKT-mTOR pathway is active, LC3B lipidation and ATG5-ATG12 complex formation decrease, thereby suppressing autophagic apoptosis (Wu et al., 2023; Hill et al., 2020). Upregulation of the TNF signaling pathway, coupled with elevated inflammatory mediators, collectively confirms that chronic inflammation is a key driver of malignant transformation in OLK.

Chemokines and cytokines such as *CSF2*, *CCL12*, *CCL20*, *IL1B*, and *CXCL3/5/6* are significantly upregulated in the tumor microenvironment, indicating that the body is extensively recruiting and activating myeloid immune cells (Arnone et al., 2021; Song et al., 2024). These cells further release additional inflammatory mediators, forming a self-amplifying positive feedback loop of inflammation. This constitutes a critical microenvironmental feature in the process of “inflammation-to-cancer conversion.” Upregulation of growth factors like *OSM*, *FGF5*, and *ARTN* directly stimulates pro-survival signaling pathways such as PI3K-Akt (Bultman, 2017), effectively delivering continuous proliferation signals to cancer cells. Concurrently, reduced expression of tumor suppressor genes *Ntrk1* and *Erbb4* weakens the cells’ intrinsic growth inhibitory mechanisms (Moore et al., 2017). Downregulation of *Mapk12* further aids cancer cells in evading stress-induced apoptosis, enhancing their survival capacity (Yang et al., 2023). Significant alterations in the expression of core basement membrane and matrix components—including *Lama1/3*, *Lamb3*, *Lamc2*, and *Col4a6/2a1*—indicate extensive matrix remodeling during carcinogenesis (Liang et al., 2020; Qin et al., 2024). This structural transformation provides the foundation for tumor invasion and metastasis. Upregulation of *Mmp3* and *Mmp9* genes directly reflects tumor cells’ ability to breach physical barriers and acquire invasive capabilities (Gao et al., 2015; Okusha et al., 2018).

Mechanistically, activation of CD46/TREM1 may exacerbates the inflammatory microenvironment by upregulating core inflammatory factors such as *Csf2*(Wang et al., 2025). Concurrently, it is associated with activation of the PI3K-Akt-mTOR pathway and suppression of autophagy (Decreased expression of LC3B/ATG5). These changes collectively lead to tumor suppressor gene inactivation, oncogene activation, and dynamic remodeling of the extracellular matrix (ECM) (as indicated by *Col4a6* expression changes), ultimately synergistically promoting OSCC proliferation, invasion, and metastasis.

This study identified differential expression of *COL4A6* between human oral cancer and rats, potentially attributable to species specificity, differences in the tumor microenvironment, and distinct gene regulatory mechanisms. Future research should further explore this issue through larger sample sizes. Ultimately, the proposed mechanistic model connecting CD46/TREM1 to OSCC progression through PI3K-AKT-mTOR-mediated autophagy suppression and effectors like *Col4a6* and *Csf2* remains to be functionally validated through targeted intervention studies in future work.

Although CD46 and TREM1 participate in distinct immune regulatory pathways—CD46 primarily influences the complement system and T cell function, while TREM1 enhances myeloid cell-mediated inflammatory responses—they exhibit significant synergy in jointly shaping the immunosuppressive tumor microenvironment. Therefore, simultaneously targeting CD46 and TREM1 may yield synergistic effects, more effectively blocking tumor immune evasion mechanisms and enhancing the body’s antitumor immune response. With deepening understanding of OSCC immunobiology and advances in targeted intervention technologies, CD46 and TREM1 hold promise as novel therapeutic targets for this disease and integration into multidisciplinary treatment strategies for OSCC. Particularly worthy of exploration is the integration of targeted therapies against these two molecules with conventional modalities such as surgery, radiotherapy, and chemotherapy to develop more personalized multimodal treatment regimens. This approach may open new avenues and perspectives for OSCC treatment.

## Data Availability

The transcriptome sequencing data generated in this study have been deposited in the Genome Sequence Archive (GSA) database at the CNCB under accession number HRA014843 (human subjects) and CRA033881 (animal subjects).
