# Supplementary material for: Exploring the interaction mechanisms of CD46/TREM1 and LC3B/ATG5 in the inflammation-cancer transformation of oral squamous cell carcinoma based on bioinformatics
Source: Front Mol Biosci. 2025 Dec 3;12:1713632. doi: 10.3389/fmolb.2025.1713632 (PMC12708265; doi:10.3389/fmolb.2025.1713632)
Supplement: Supplementary file 3 [file Supplementaryfile1.docx]

**Supplementary Figure S1**. Volcano plots and clustering heatmaps of DEGs across groups. (A-C) Volcano plots display DEGs between specified groups, with the control group indicated second: (A) INF versus NC, (B) OLK versus INF, (C) OSCC versus OLK. Red dots, upregulated genes; blue dots, downregulated genes (relative to control); gray dots, non-significant genes. (D-F) Clustering heatmaps of DEGs for the same comparisons shown in A-C. Columns represent individual samples; rows represent genes. The color scale indicates expression levels, with red and blue denoting high and low expression, respectively.





**Supplementary Figure S2**. Volcano plots and clustering heatmaps of DEGs across groups. (A, B) Volcano plots display DEGs between specified groups, with the control group indicated second: (A) NC versus OLK, (B) OLK versus OSCC. Red dots, upregulated genes; blue dots, downregulated genes (relative to control); gray dots, non-significant genes. (C, D) Clustering heatmaps of DEGs for the same comparisons shown in A and B. Columns represent individual samples; rows represent genes. The color scale indicates expression levels, with red and blue denoting high and low expression, respectively.
